# Effective alignment of RNA pseudoknot structures using partition function posterior log-odds scores

**DOI:** 10.1186/s12859-015-0464-9

**Published:** 2015-02-06

**Authors:** Yang Song, Lei Hua, Bruce A Shapiro, Jason TL Wang

**Affiliations:** 1 Bioinformatics Program, Department of Computer Science, New Jersey Institute of Technology, University Heights, Newark, New Jersey 07102 USA; 2 Computational RNA Structure Group, Center for Cancer Research, Basic Research Laboratory, National Cancer Institute, Frederick, Maryland 21702 USA

**Keywords:** RNA secondary structure including pseudoknots, Structural alignment, Dynamic programming algorithm

## Abstract

**Background:**

RNA pseudoknots play important roles in many biological processes. Previous methods for comparative pseudoknot analysis mainly focus on simultaneous folding and alignment of RNA sequences. Little work has been done to align two known RNA secondary structures with pseudoknots taking into account both sequence and structure information of the two RNAs.

**Results:**

In this article we present a novel method for aligning two known RNA secondary structures with pseudoknots. We adopt the partition function methodology to calculate the posterior log-odds scores of the alignments between bases or base pairs of the two RNAs with a dynamic programming algorithm. The posterior log-odds scores are then used to calculate the expected accuracy of an alignment between the RNAs. The goal is to find an optimal alignment with the maximum expected accuracy. We present a heuristic to achieve this goal. The performance of our method is investigated and compared with existing tools for RNA structure alignment. An extension of the method to multiple alignment of pseudoknot structures is also discussed.

**Conclusions:**

The method described here has been implemented in a tool named RKalign, which is freely accessible on the Internet. As more and more pseudoknots are revealed, collected and stored in public databases, we anticipate a tool like RKalign will play a significant role in data comparison, annotation, analysis, and retrieval in these databases.

**Electronic supplementary material:**

The online version of this article (doi:10.1186/s12859-015-0464-9) contains supplementary material, which is available to authorized users.

## Background

RNA pseudoknots are formed by pairing bases on single-stranded loops, such as hairpin and internal loops, with bases outside the loops [1,2]. They are often mingled with other RNA tertiary motifs [3], and are also found in non-coding RNAs [4,5]. RNA pseudoknots, with diverse functions [6,7], play important roles in many biological processes [8,9]; for example, they are required for telomerase activity [7], and have been shown to regulate the efficiency of ribosomal frameshifting in viruses [10].

Analysis and detection of RNA pseudoknots has been an active area of research. Many published articles in this area were focused on pseudoknot alignment [11-14]. In this paper, we present a new approach, called RKalign, for RNA pseudoknot alignment. RKalign accepts as input two pseudoknotted RNAs where each RNA has both sequence data (i.e. nucleotides or bases) and structure data (i.e. base pairs), and produces as output an alignment between the two pseudoknotted RNAs. The structure data of a pseudoknotted RNA can be obtained from the literature or public databases [15-18].

RKalign adopts the partition function methodology to calculate the posterior probabilities or log-odds scores of structural alignments. The idea of using posterior probabilities to align biomolecules originated from [19,20] where the partition function methodology was employed to calculate the posterior probabilities of protein sequence alignments. Similar techniques were proposed by Do et al. [21] where the authors used hidden Markov models (HMMs) to calculate the posterior probabilities. Will et al. [22] extended the idea of [19-21] to structure-based multiple RNA alignment where the authors calculated partition functions inside and outside of subsequence pairs on two pseudoknot-free RNAs. Here, we further extend this idea to pseudoknot alignment.

Several tools are available for RNA sequence-structure alignment [23-25]. These tools do not deal with pseudoknots. Mohl et al. [26] proposed a method to perform sequence-structure alignment for RNA pseudoknots. The authors set up a pipeline for combining alignment and prediction of pseudoknots, and showed experimentally the effectiveness of this pipeline in pseudoknot structure annotation. Han et al. [27] decomposed embedded pseudoknots into simple pseudoknots and aligned them recursively. Yoon [28] used a profile-HMM to establish sequence alignment constraints, and incorporated these constraints into an algorithm for aligning RNAs with pseudoknots. Wong et al. [29] identified the pseudoknot type of a given structure and developed dynamic programming algorithms for structural alignments of different pseudoknot types. Huang et al. [4] applied a tree decomposition algorithm to search for non-coding RNA pseudoknot structures in genomes.

The above methods were concerned with aligning a pseudoknot structure with a sequence or genome. Through the alignment, the sequence is folded and its structure is predicted. Xu et al. [11] presented a different method, called RNA Sampler, which can simultaneously fold and align two or multiple RNA sequences considering pseudoknots without known structures. Similar techniques were implemented in DAFS [12] and SimulFold [13]. Additional methods can be found in the CompaRNA web server [30]. In contrast to these methods, which perform alignment and folding at the same time, RKalign aims to align two known RNA pseudoknot structures where the structures are obtained from existing databases [15-17]. As more pseudoknot structures become available in these databases, a tool like RKalign will be useful in performing data analysis in the repositories.

There are two groups of algorithms which are also capable of aligning two known RNA structures. The first group is concerned with aligning two RNA three-dimensional (3D) structures, possibly containing pseudoknots. Ferre et al. [31] presented a dynamic programming algorithm by taking into account nucleotide, dihedral angle and base-pairing similarities. Capriotti and Marti-Renom [32] developed a program to align two RNA 3D structures based on a unit-vector root-mean-square approach. Chang et al. [33] and Wang et al. [34] employed a structural alphabet of different nucleotide conformations to align RNA 3D structures. Hoksza and Svozil [35] developed a pairwise comparison method based on 3D similarity of generalized secondary structure units. Rahrig et al. [36] presented the R3D Align tool for performing global pairwise alignment of RNA 3D structures using local superpositions. He et al. [37] developed the RASS web server for comparing RNA 3D structures using both sequence and 3D structure information. The above methods and tools were mainly designed for aligning two RNA tertiary structures by considering their geometric properties and torsion angles. In contrast, RKalign is used to align two RNA secondary structures with pseudoknots.

The second group of algorithms is concerned with aligning two RNA secondary structures without pseudoknots. These algorithms employed general edit-distance alignment [38] or tree matching techniques [39-41]. Jiang et al. [42] developed an approximation algorithm for aligning a pseudoknot-free structure with a pseudoknotted structure. Our work differs from Jiang et al.’s work in that we focus on the alignment of two pseudoknotted structures. Furthermore, we use the partition function methodology whereas Jiang et al. adopted a general edit-distance approach to the structural alignment.

The method that is most closely related to ours is an option offered by the CARNA tool [14]. Like RKalign, this option is able to accept two known RNA secondary structures with pseudoknots, and produce an alignment between the two RNA structures. This option employs constraint programming techniques with a branch and bound scheme. It gradually refines solutions until the best solution is found. To understand the relative performance of the two tools, we perform extensive experiments to compare RKalign with CARNA using different datasets.

## Methods

In this section, we present algorithmic details of RKalign. To align two RNA pseudoknot structures *A* and *B*, we adopt the partition function methodology to calculate the posterior probabilities or log-odds scores of the alignments between bases or base pairs in *A* and *B* respectively. After calculating the posterior log-odds scores, we then compute the expected accuracy of an alignment between structure *A* and structure *B*. The goal is to find an optimal alignment between *A* and *B* where the alignment has the maximum expected accuracy. We will present a heuristic to achieve this goal.

### Definitions and notation

Suppose (*i*, *j*) is a base pair of pseudoknot structure *A* and (*p*, *q*) is a base pair of pseudoknot structure *B*. We use *score*((*i*, *j*), (*p*, *q*)) to represent the score of aligning (*i*, *j*) with (*p*, *q*) where the score is obtained from the log-odds RIBOSUM matrix [43]. The use of this scoring matrix permits RKalign to determine the similarity between pseudoknot structures that contain compensatory base changes. With this scoring matrix, RKalign is able to handle non-canonical base pairs. Aligning a single base with a base pair is prohibited by RKalign.

Suppose structure *A* has *m* nucleotides, i.e. the length of *A* is *m*, and structure *B* has *n* nucleotides, i.e. the length of *B* is *n*. We use *A*[*c*_1_, *c*_2_] where 1 ≤ *c*_1_ ≤ *c*_2_ ≤ *m* to represent the portion of *A* that begins at position *c*_1_ and ends at position *c*_2_ inclusively. We use *B*[*d*_1_, *d*_2_] where 1 ≤ *d*_1_ ≤ *d*_2_ ≤ *n* to represent the portion of *B* that begins at position *d*_1_ and ends at position *d*_2_ inclusively. We use *A*[*c*] to represent the nucleotide and secondary structure at position *c* of *A*, and *B*[*d*] to represent the nucleotide and secondary structure at position *d* of *B*.

### Partition function computation

Suppose (*i*, *j*) ∈ *A* is aligned with (*p*, *q*) ∈ *B.* Let *Z*_*c*,*d*_ (${Z}_{c,\ d}^{\prime }$ respectively) represent the partition function of all alignments between *A*[1, *c*] (*A*[*c*, *m*] respectively) and *B*[1, *d*] (*B*[*d*, *n*] respectively). Let ${Z^{\prime}}_{c,d}^{\prime }$ represent the partition function of all alignments between *A*[*i +* 1, *c*] and *B*[*p +* 1, *d*]. We focus on the case in which both (*i*, *j*) and (*p*, *q*) are base pairs. The case for aligning single bases is simpler, and thus omitted.

First, we show how to calculate *Z*_*c*,*d*_ where 1 ≤ *c* < *i* and 1 ≤ *d* < *p*. There are three cases to be considered: (i) *A*[*c*] is aligned with *B*[*d*]; (ii) *B*[*d*] is aligned to a gap; and (iii) *A*[*c*] is aligned to a gap. Let ${Z}_{c,d}^M$ represent the partition function of all alignments between *A*[1, *c*] and *B*[1, *d*] where *A*[*c*] is aligned with *B*[*d*]. Let ${Z}_{c,d}^E$ represent the partition function of all alignments between *A*[1, *c*] and *B*[1, *d*] where *B*[*d*] is aligned to a gap. Let ${Z}_{c,d}^F$ represent the partition function of all alignments between *A*[1, *c*] and *B*[1, *d*] where *A*[*c*] is aligned to a gap. Then *Z*_*c*,*d*_ can be calculated by Equation (1).

1 $$ {Z}_{c,d}={Z}_{c,d}^M+{Z}_{c,d}^E+{Z}_{c,d}^F $$

We ignore and skip the computation of *Z*_*c*,*d*_ when *A*[*c*] or *B*[*d*] is the left base of some base pair. If *A*[*c*] (*B*[*d*], respectively) is a single base and *B*[*d*] (*A*[*c*], respectively) is the right base of some base pair, ${Z}_{c,d}^M=0$. Otherwise, let *A*[*c*] be the right base of some base pair (*x*, *c*) and let *B*[*d*] be the right base of some base pair (*y*, *d*)*.* Following [20], ${Z}_{c,d}^M$ can be calculated by Equation (2).

2 $$ {Z}_{c,d}^M={Z}_{c-1,d-1}{e}^{\frac{score\left(\left(x,\ c\right),\ \left(y,\ d\right)\right)}{T}} $$

Here *T* is a constant, and *score*((*x*, *c*), (*y*, *d*)) is obtained from the RIBOSUM85-60 matrix [43]. Thus, the partition function ${Z}_{c,d}^M$ can be computed recursively by dynamic programming as follows:

3 $$ {Z}_{c,d}^M=\left({Z}_{c-1,d-1}^M+{Z}_{c-1,d-1}^E+{Z}_{c-1,d-1}^F\right){e}^{\frac{score\left(\left(x,\ c\right),\ \left(y,\ d\right)\right)}{T}} $$

When calculating ${Z}_{c,d}^E$, since *B*[*d*] is aligned to a gap, we know that *A*[*c*] must be aligned with *B*[*d*-1]. Therefore,

4 $$ {Z}_{c,d}^E={Z}_{c,d-1}{e}^{\frac{score\left(-,\left(y,d\right)\right)}{T}} $$

where *score*(−, (*y*, *d*)) is the gap penalty value obtained by aligning base pair (*y*, *d*) to gaps. Thus,

5 $$ {Z}_{c,d}^E=\left({Z}_{c,d-1}^M+{Z}_{c,\ d-1}^E+{Z}_{c,d-1}^F\right){e}^{\frac{score\left(-,\left(y,d\right)\right)}{T}} $$

When calculating ${Z}_{c,d}^F$, since *A*[*c*] is aligned to a gap, *B*[*d*] must be aligned with *A*[*c*-1]. Therefore,

6 $$ {Z}_{c,d}^F={Z}_{c-1,d}{e}^{\frac{score\left(\left(x,\ c\right),-\right)}{T}} $$

where *score*((*x*, *c*),−) is the gap penalty value obtained by aligning base pair (*x*, *c*) to gaps. Thus,

7 $$ {Z}_{c,d}^F=\left({Z}_{c-1,d}^M+{Z}_{c-1,d}^E+{Z}_{c-1,d}^F\right){e}^{\frac{score\left(\left(x,\ c\right),-\right)}{T}} $$

Next, we show how to calculate ${Z}_{c,\ d}^{\prime }$ where *j* < *c* ≤ *m* and *q* < *d* ≤ *n*. There are three cases to be considered: (i) *A*[*c*] is aligned with *B*[*d*]; (ii) *B*[*d*] is aligned to a gap; and (iii) *A*[*c*] is aligned to a gap. Let ${Z^{\prime}}_{c,d}^M$ represent the partition function of all alignments between *A*[*c*, *m*] and *B*[*d*, *n*] where *A*[*c*] is aligned with *B*[*d*]. Let ${Z^{\prime}}_{c,d}^E$ represent the partition function of all alignments between *A*[*c*, *m*] and *B*[*d*, *n*] where *B*[*d*] is aligned to a gap. Let ${Z^{\prime}}_{c,d}^F$ represent the partition function of all alignments between *A*[*c*, *m*] and *B*[*d*, *n*] where *A*[*c*] is aligned to a gap. Then ${Z}_{c,\ d}^{\prime }$ can be calculated by Equation (8).

8 $$ {Z}_{c,\ d}^{\prime }={Z^{\prime}}_{c,d}^M+{Z^{\prime}}_{c,d}^E+{Z^{\prime}}_{c,d}^F $$

We ignore the computation of ${Z}_{c,\ d}^{\prime }$ when *A*[*c*] or *B*[*d*] is the right base of some base pair. If *A*[*c*] (*B*[*d*], respectively) is a single base and *B*[*d*] (*A*[*c*], respectively) is the left base of some base pair, ${Z^{\prime}}_{c,d}^M=0$. Otherwise, let *A*[*c*] be the left base of some base pair (*c*, *x*) and let *B*[*d*] be the left base of some base pair (*d*, *y*). Following [20], ${Z^{\prime}}_{c,d}^M$ can be calculated by Equation (9).

9 $$ \begin{array}{l}{Z^{\prime}}_{c,d}^M={Z}_{c+1,d+1}^{\prime }{e}^{\frac{score\left(\left(c,\ x\right),\ \left(d,\ y\right)\right)}{T}}\hfill \\ {}\kern3.5em =\left({Z^{\prime}}_{c+1,d+1}^M+{Z^{\prime}}_{c+1,d+1}^E+{Z^{\prime}}_{c+1,d+1}^F\right){e}^{\frac{score\left(\left(c,\ x\right),\ \left(d,\ y\right)\right)}{T}}\hfill \end{array} $$

When calculating ${Z^{\prime}}_{c,d}^E$, since *B*[*d*] is aligned to a gap, *A*[*c*] must be aligned with *B*[*d* + 1]. Therefore,

10 $$ \begin{array}{l}{Z^{\prime}}_{c,d}^E={Z}_{c,d+1}^{\prime }{e}^{\frac{score\left(-,\left(d,y\right)\right)}{T}}\hfill \\ {}\kern3.5em =\left({Z^{\prime}}_{c,d+1}^M+{Z^{\prime}}_{c,d+1}^E+{Z^{\prime}}_{c,d+1}^F\right){e}^{\frac{score\left(-,\left(d,y\right)\right)}{T}}\hfill \end{array} $$

When calculating ${Z^{\prime}}_{c,d}^F$, since *A*[*c*] is aligned to a gap, *B*[*d*] must be aligned with *A*[*c* + 1]. Therefore,

11 $$ \begin{array}{l}\begin{array}{l}{Z^{\prime}}_{c,d}^F={Z}_{c+1,d}^{\prime }{e}^{\frac{score\left(\left(c,x\right),-\right)}{T}}\hfill \\ {}\kern3.5em =\left({Z^{\prime}}_{c+1,d}^M+{Z^{\prime}}_{c+1,d}^E+{Z^{\prime}}_{c+1,d}^F\right){e}^{\frac{score\left(\left(c,x\right),-\right)}{T}}\hfill \end{array}\\ {}\end{array} $$

Finally, we show how to calculate ${Z^{\prime}}_{c,d}^{\prime }$ where *i* < *c* < *j* and *p* < *d* < *q*. There are three cases to be considered: (i) *A*[*c*] is aligned with *B*[*d*]; (ii) *B*[*d*] is aligned to a gap; and (iii) *A*[*c*] is aligned to a gap. Let ${{Z^{\prime}}^{\prime}}_{c,d}^M$ represent the partition function of all alignments between *A*[*i +* 1, *c*] and *B*[*p +* 1, *d*] where *A*[*c*] is aligned with *B*[*d*]. Let ${{Z^{\prime}}^{\prime}}_{c,d}^E$ represent the partition function of all alignments between *A*[*i +* 1, *c*] and *B*[*p +* 1, *d*] where *B*[*d*] is aligned to a gap. Let ${{Z^{\prime}}^{\prime}}_{c,d}^F$ represent the partition function of all alignments between *A*[*i +* 1, *c*] and *B*[*p +* 1, *d*] where *A*[*c*] is aligned to a gap. Then ${Z^{\prime}}_{c,d}^{\prime }$ can be calculated by Equation (12).

12 $$ {Z^{\prime}}_{c,d}^{\prime }={{Z^{\prime}}^{\prime}}_{c,d}^M + {{Z^{\prime}}^{\prime}}_{c,d}^E+{{Z^{\prime}}^{\prime}}_{c,d}^F $$

We ignore the computation of ${Z^{\prime}}_{c,d}^{\prime }$ when *A*[*c*] or *B*[*d*] is the left base of some base pair. If *A*[*c*] (*B*[*d*], respectively) is a single base and *B*[*d*] (*A*[*c*], respectively) is the right base of some base pair, ${{Z^{\prime}}^{\prime}}_{c,d}^M=0$. Otherwise, let *A*[*c*] be the right base of some base pair (*x*, *c*) and let *B*[*d*] be the right base of some base pair (*y*, *d*). If *x* < *i* + 1 or *y* < *p* + 1, we ignore and skip the computation of ${Z^{\prime}}_{c,d}^{\prime }$ *.* We consider only the case where *x* ≥ *i* + 1 and *y* ≥ *p* + 1. Following [20], ${{Z^{\prime}}^{\prime}}_{c,d}^M$ can be calculated by Equation (13).

13 $$ \begin{array}{l}\ \\ {}\begin{array}{l}{{Z^{\prime}}^{\prime}}_{c,d}^M={Z^{\prime}}_{c-1,\ d-1}^{\prime }{e}^{\frac{score\left(\left(x,\ c\right),\ \left(y,\ d\right)\right)}{T}}\hfill \\ {}\kern3.5em =\left({{Z^{\prime}}^{\prime}}_{c-1,d-1}^M+{{Z^{\prime}}^{\prime}}_{c-1,d-1}^E+{{Z^{\prime}}^{\prime}}_{c-1,d-1}^F\right){e}^{\frac{score\left(\left(x,\ c\right),\ \left(y,\ d\right)\right)}{T}}\hfill \end{array}\end{array} $$

When calculating ${{Z^{\prime}}^{\prime}}_{c,d}^E$, since *B*[*d*] is aligned to a gap, *A*[*c*] must be aligned with *B*[*d*-1]. Therefore,

14 $$ \begin{array}{l}{{Z^{\prime}}^{\prime}}_{c,d}^E={Z^{\prime}}_{c,\ d-1}^{\prime }{e}^{\frac{score\left(-,\ \left(y,\ d\right)\right)}{T}}\hfill \\ {}\kern3.5em =\left({{Z^{\prime}}^{\prime}}_{c,d-1}^M+{{Z^{\prime}}^{\prime}}_{c,d-1}^E+{{Z^{\prime}}^{\prime}}_{c,d-1}^F\right){e}^{\frac{score\left(-,\ \left(y,\ d\right)\right)}{T}}\hfill \end{array} $$

When calculating ${{Z^{\prime}}^{\prime}}_{c,d}^F$, since *A*[*c*] is aligned to a gap, *B*[*d*] must be aligned with *A*[*c*-1]. Therefore,

15 $$ \begin{array}{l}{{Z^{\prime}}^{\prime}}_{c,d}^F={Z^{\prime}}_{c-1,\ d}^{\prime }{e}^{\frac{score\left(\left(x,c\right),-\right)}{T}}\hfill \\ {}\kern3.5em =\left({{Z^{\prime}}^{\prime}}_{c-1,d}^M+{{Z^{\prime}}^{\prime}}_{c-1,d}^E+{{Z^{\prime}}^{\prime}}_{c-1,d}^F\right){e}^{\frac{score\left(\left(x,\ c\right),-\right)}{T}}\hfill \end{array} $$

### Calculation of posterior log-odds scores

There are four cases to be considered when calculating the posterior probability or log-odds score of aligning base pair (*i*, *j*) of structure *A* with base pair (*p*, *q*) of structure *B*, denoted by *Prob*((*i*, *j*) ~ (*p*, *q*)).

#### Case 1

Base pair (*i*, *j*) doesn’t cross another base pair and (*p*, *q*) doesn’t cross another base pair. That is, for any base pair (*u*, *v*), *i* < *v* < *j* if and only if *i* < *u* < *j*. Furthermore, for any base pair (*x*, *y*), *p* < *y* < *q* if and only if *p* < *x* < *q*. Consequently, the alignment between structure *A* and structure *B* can be divided into the following three parts: (i) the alignment between *A*[1, *i* – 1] and *B*[1, *p* – 1]; (ii) the alignment between *A*[*i* + 1, *j* – 1] and *B*[*p* + 1, *q* – 1]; and (iii) the alignment between *A*[*j* + 1, *m*] and *B*[*q* + 1, *n*]. Following [20] we get

16 $$ Prob\left(\left(i,j\right)\sim \left(p,q\right)\right)=\frac{Z_{i-1,p-1}{Z^{\prime}}_{j-1,q-1}^{\prime }{Z}_{j+1,q+1}^{\prime }}{Z_{m,n}}{e}^{\frac{score\left(\left(i,j\right),\left(p,q\right)\right)}{T}} $$

#### Case 2

Base pair (*i*, *j*) crosses another base pair whereas (*p*, *q*) doesn’t cross another base pair. That is, there exists a base pair (*u*, *v*) in *A* such that (i) *i* < *v* < *j* and *u* < *i*, or (ii) *i* < *u* < *j* and *v* > *j*. Furthermore, for any base pair (*x*, *y*), *p* < *y* < *q* if and only if *p* < *x* < *q*. In this case, (*i*, *j*) crosses (*u*, *v*), which forms a pseudoknot in structure *A*, while (*p*, *q*) doesn’t form a pseudoknot in structure *B*.

When (i) is true, since *u* < *i*, we have 1 ≤ *u* ≤ *i* − 1. Furthermore, since *v* > *i* > *i* − 1, (*u*, *v*) is ignored when calculating *Z*_*i* − 1,*p* − 1_ in Equation (16). In addition, since *u* < *i* < *i* + 1, (*u*, *v*) is ignored when calculating ${Z^{\prime}}_{j-1,q-1}^{\prime }$ in Equation (16). Base pair (*u*, *v*) will be considered when calculating *Prob* ((*u*, *v*) ~ (*p*, *q*)). Thus, our algorithm doesn’t miss the calculation of the posterior log-odds score of aligning any two base pairs from structure *A* and structure *B* respectively.

When (ii) is true, since *v* > *j*, we have *j* + 1 ≤ *v* ≤ *m*. Furthermore, since *u* < *j* < *j* + 1, (*u*, *v*) is ignored when calculating ${Z}_{j+1,q+1}^{\prime }$ in Equation (16). Base pair (*u*, *v*) will be considered when calculating *Prob* ((*u*, *v*) ~ (*p*, *q*)).

#### Case 3

Base pair (*p*, *q*) crosses another base pair whereas (*i*, *j*) doesn’t cross another base pair. This case is similar to Case 2 above.

#### Case 4

Base pair (*i,j*) crosses another base pair and (*p,q*) also crosses another base pair. That is, there exists a base pair (*u,v*) in *A* such that (i) *i* < *v* < *j* and *u* < *i*, or (ii) *i* < *u* < *j* and *v* > *j*. Furthermore, there exists a base pair (*x*, *y*) in *B* such that (iii) *p* < *y* < *q* and *x* < *p*, or (iv) *p* < *x* < *q* and *y* > *q*. In this case, (*i,j*) crosses (*u,v*), which forms a pseudoknot in structure *A*. Furthermore (*p,q*) crosses (*x,y*), which also forms a pseudoknot in structure *B*.

When (i) and (iii) are true, (*u*, *v*) is ignored when calculating *Z*_*i* − 1,*p* − 1_ and ${Z^{\prime}}_{j-1,q-1}^{\prime }$ as discussed in Case 2 (i). Moreover, (*x*, *y*) is also ignored when calculating *Z*_*i* − 1,*p* − 1_ and ${Z^{\prime}}_{j-1,q-1}^{\prime }$ (Case 3). When (i) and (iv) are true, (*u*, *v*) is ignored when calculating *Z*_*i* − 1,*p* − 1_ and ${Z^{\prime}}_{j-1,q-1}^{\prime }$ (Case 2 (i)); (*x*, *y*) is also ignored when calculating ${Z}_{j+1,q+1}^{\prime }$ (Case 3). When (ii) and (iii) are true, (*u*, *v*) is ignored when calculating *Z* ^'^_*j* + 1,*q* + 1_ (Case 2 (ii)); (*x*, *y*) is also ignored when calculating *Z*_*i* − 1,*p* − 1_ and ${Z^{\prime}}_{j-1,q-1}^{\prime }$ (Case 3). When (ii) and (iv) are true, (*u*, *v*) is ignored when calculating ${Z}_{j+1,q+1}^{\prime }$ (Case 2 (ii)); (*x*, *y*) is also ignored when calculating ${Z}_{j+1,q+1}^{\prime }$ (Case 3).

When both (*i*, *j*) and (*p*, *q*) are single bases, i.e. *i* = *j* and *p* = *q*, the value of ${Z^{\prime}}_{j-1,q-1}^{\prime }$ in Equation (16) is defined as 1, and we use the same formula in Equation (16) to calculate *Prob*((*i*, *j*) ~ (*p*, *q*)).

From the above discussions, Equation (16) can be used to calculate the posterior log-odds score of aligning two bases or base pairs with a dynamic programming algorithm. Furthermore, the algorithm doesn’t miss the calculation of the posterior log-odds score of aligning any two bases or base pairs from structure *A* and structure *B* respectively.

### Pairwise alignment

Let *a*_*A,B*_ be an alignment between structure *A* and structure *B*. The expected accuracy of *a*_*A,B*,_ denoted $\overline{Accu}\left({a}_{A,B}\right)$, is defined as follows [21]:

17 $$ \overline{Accu}\left({a}_{A,B}\right)=\frac{{\displaystyle {\sum}_{\left(\left(i,j\right)\sim \left(p,q\right)\in {a}_{A,B}\right)} Prob}\left(\left(i,j\right)\sim \left(p,q\right)\right)}{max\left\{h,\left.k\right\}\right.} $$

where ((*i*, *j*) ~ (*p*, *q*) ∈ *a*_*A*,*B*_) means (*i*, *j*) ∈ *A* is aligned with (*p*, *q*) ∈ *B* in *a*_*A,B*_, *Prob*((*i*, *j*) ~ (*p*, *q*)) is the posterior log-odds score of aligning (*i*, *j*) ∈ *A* with (*p*, *q*) ∈ *B* as defined in Equation (16), and *h* (*k* respectively) is the number of single bases plus the number of base pairs in *A* (*B* respectively).

An optimal alignment between structure *A* and structure *B* is an alignment with the maximum expected accuracy. We present here a heuristic to find a (sub)optimal alignment. From the previous subsection, we are able to construct the posterior log-odds score matrix for aligning structure *A* with structure *B* where the matrix contains *Prob*((*i*, *j*) ~ (*p*, *q*)) for all (*i*, *j*) ∈ *A* and (*p*, *q*) ∈ *B*. Our heuristic is an iterative procedure. In the first step, we select two bases or base pairs with the largest score from this matrix to build the first alignment line between *A* and *B* where the alignment line connects the selected bases or base pairs. Then, we select the second largest score from the matrix to construct the next alignment line provided that the newly constructed alignment line satisfies the following two constraints:

1. A base (base pair, respectively) can be aligned with at most one base (base pair, respectively).
2. The newly constructed alignment lines do not cross the alignment lines built in the previous steps. Specifically, suppose (*i*, *j*) is aligned with (*p*, *q*) and (*i* ', *j* ') is aligned with (*p* ', *q* '). The alignment lines between (*i*, *j*) and (*p*, *q*) do not cross the alignment lines between (*i* ', *j* ') and (*p* ', *q* ') if and only if the following conditions hold: (i) *i* ' < *i* iff *p* ^'^ < *p*, (ii) *i* < *i* ' < *j* iff *p* < *p* ' < *q*, (iii) *i* ' > *j* iff *p* ' > *q*, (iv) *j* ' < *i* iff *q* ' < *p*, (v) *i* < *j* ' < *j* iff *p* < *q* ' < *q* and (vi) *j* ' > *j* iff *q* ' > *q*.

If the newly constructed alignment line violates the above constraints, it is discarded. We repeat the above steps until the smallest posterior log-odds score in the matrix is considered. If there are still bases or base pairs that are not aligned yet, these remaining bases or base pairs are aligned to gaps.

### Time and space complexity

In calculating *Prob*((*i*, *j*) ~ (*p*, *q*)), we need to compute *Z*_*i* − 1,*p* − 1_, ${Z^{\prime}}_{j-1,q-1}^{\prime }$ and *Z* ^'^_*j* + 1,*q* + 1_; cf. Equation (16). Computing *Z*_*i* − 1,*p* − 1_, ${Z^{\prime}}_{j-1,q-1}^{\prime }$ and ${Z}_{j+1,q+1}^{\prime }$ requires *O*(*mn*) time. Since we need to calculate *Prob*((*i*, *j*) ~ (*p*, *q*)) for all (*i*, *j*) ∈ *A* and (*p*, *q*) ∈ *B*, the time complexity of the pairwise alignment algorithm is *O*(*m* ^2^ *n* ^2^). At any moment, we maintain a two-dimensional matrix for storing *Z*_*i* − 1,*p* − 1_, ${Z^{\prime}}_{j-1,q-1}^{\prime }$ and ${Z}_{j+1,q+1}^{\prime }$, which requires *O*(*mn*) space. Since the total number of bases and base pairs in structure *A* (*B* respectively) is at most *m* (*n* respectively), we use a two-dimensional matrix to store *Prob*((*i*, *j*) ~ (*p*, *q*)), which also requires *O*(*mn*) space. Thus, the space complexity of the algorithm is *O*(*mn*). Notice that the time complexity derived here is a very pessimistic upper bound since in calculating the partition functions, some base pairs are ignored as described in the previous subsections. During our experiments, we tested over 200 alignments and the running times of our algorithm ranged from 16 ms to roughly 7 minutes, where the lengths of the aligned structures ranged from 22 nt to 1,553 nt.

### Experimental design

#### Datasets

RKalign is implemented in Java. The program accepts as input two pseudoknotted RNAs where each RNA has both sequence data (i.e. nucleotides or bases) and structure data (i.e. base pairs), and produces as output an alignment between the two pseudoknotted RNAs. Popular benchmark datasets such as BRAliBase [44], RNase P [45] and Rfam [46] are not suitable for testing RKalign. The reason is that BRAliBase contains only sequence information, while RNase P and Rfam contain consensus structures of multiple sequence alignments rather than alignments of individual structures of RNAs. As a consequence, we manually created two datasets for testing RKalign and comparing it with related alignment methods.

The first dataset, denoted Dataset1, contains 38 RNA pseudoknot structures chosen from the PDB [16] and RNA STRAND [15] (see Additional file 1: Table S1). These RNAs were selected in such a way that they have a wide range of sequence lengths. Each three-dimensional (3D) molecule in this dataset was taken from the PDB. The secondary structure of the 3D molecule was obtained with RNAview [47], retrieved from RNA STRAND. The second dataset, denoted Dataset2, contains 36 RNA pseudoknot structures chosen from PseudoBase [17,18] (see Additional file 1: Table S2). As in the first dataset, the RNA molecules in the second dataset have a wide range of sequence lengths. The pseudoknots in these datasets can be broadly classified into two types: H-type and recursive pseudoknots [8,29]. There are 12 H-type pseudoknots and 26 recursive pseudoknots in Dataset1. There are 22 H-type pseudoknots and 14 recursive pseudoknots in Dataset2.

#### Alignment quality

A good structural alignment tends to align a base pair with another base pair rather than with two single bases [35,36]. We therefore use the base_mismatch ratio to assess the quality of an alignment. A base mismatch occurs when a single base is aligned with the left or right base of a base pair or when a nucleotide is aligned to a gap. The base_mismatch ratio of an alignment *a*_*A,B*_ between structure *A* and structure *B* is defined as the number of base mismatches in *a*_*A,B*_ divided by the total number of alignment lines in *a*_*A,B*_, multiplied by 100%. Statistically significant performance differences between alignment methods are calculated using Wilcoxon signed rank tests [48], which are commonly used for comparing alignment programs [49-51]. As in [49-51] we consider p-values below 0.05 to be statistically significant.

## Results

We conducted a series of experiments to evaluate the performance of RKalign and compare it with related methods, where the performance measure used was the base_mismatch ratio. In the first experiment, we selected 106 pairs of RNA pseudoknot structures from Dataset1 and applied our method to aligning the two molecules in each pair. The two molecules in a pair belonged to the same pseudoknot type, as it is biologically meaningless to align RNA molecules that lack consensus [35,52]. The average base_mismatch ratio calculated by RKalign for the selected 106 pairs was 34.84%, compared to the average base_mismatch ratio, 78.53%, for all pairs of molecules in Dataset1.

In addition, we also ran CARNA [14], RNA Sampler [11], DAFS [12], R3D Align [53] and RASS [37] on the 106 pairs of molecules. The CARNA tool was chosen because an option of the tool is closely related to RKalign, both of which can align known pseudoknot structures. RNA Sampler and DAFS were chosen because they are widely used tools capable of simultaneously folding and aligning RNA sequences considering pseudoknots without known structures. When running these two tools, the structure information in Dataset1 was ignored and only the sequence data was used as the input of the tools. R3D Align and RASS were chosen because they are state-of-the-art RNA 3D alignment programs; furthermore, like RKalign, R3D Align and RASS output the entire alignment of two RNA structures. Since R3D Align and RASS accept 3D structures as input whereas RKalign and CARNA accept bases and base pairs as input, we used the PDB files in Dataset1 as the input for R3D Align and RASS while using the corresponding RNA STRAND entries in Dataset1 as the input for RKalign and CARNA. The 106 pairwise alignments produced by RKalign can be found in Additional file 2.

**Figure 1:**
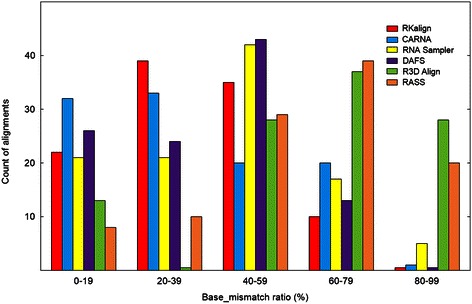
**Histogram for the base_mismatch ratios yielded by RKalign, CARNA, RNA Sampler, DAFS, R3D Align and RASS.** Histograms for the base_mismatch ratios of the alignments produced by RKalign, CARNA, RNA Sampler, DAFS, R3D Align and RASS respectively on the 106 structure pairs selected from Dataset1. Buckets on the x-axis are defined by equal-width ranges 0 to19, 20 to 39, 40 to 59, 60 to 79, and 80 to 99 (rounded down to the nearest whole number). These histograms show the distribution of the base_mismatch ratios of the alignments produced by the six tools.

**Figure 2:**
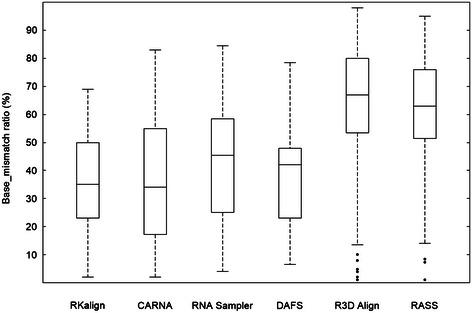
**Boxplot for the base_mismatch ratios of RKalign, CARNA, RNA Sampler, DAFS, R3D Align and RASS.** Boxplots for the base_mismatch ratios of the alignments produced by RKalign, CARNA, RNA Sampler, DAFS, R3D Align and RASS respectively on the 106 structure pairs selected from Dataset1. The median of the base_mismatch ratios yielded by RKalign (CARNA, RNA Sampler, DAFS, R3D Align, RASS, respectively) is 35.29% (34.38%, 45.86%, 41.99%, 67.57%, 63.29%, respectively).

Figure 1 presents histograms for the base_mismatch ratios of the six tools. Figure 2 presents boxplots for the base_mismatch ratios of the six tools. These figures show the distribution of the base_mismatch ratios for the six tools. RKalign and CARNA were not statistically different according to a Wilcoxon signed rank test (p > 0.05). On the other hand, they both were significantly better than the other four tools according to the Wilcoxon signed rank test (p < 0.05). It was observed that the structures predicted by RNA Sampler and DAFS might not be correct. Consequently, there were many base mismatches with respect to the known structures in the alignments.

**Figure 3:**
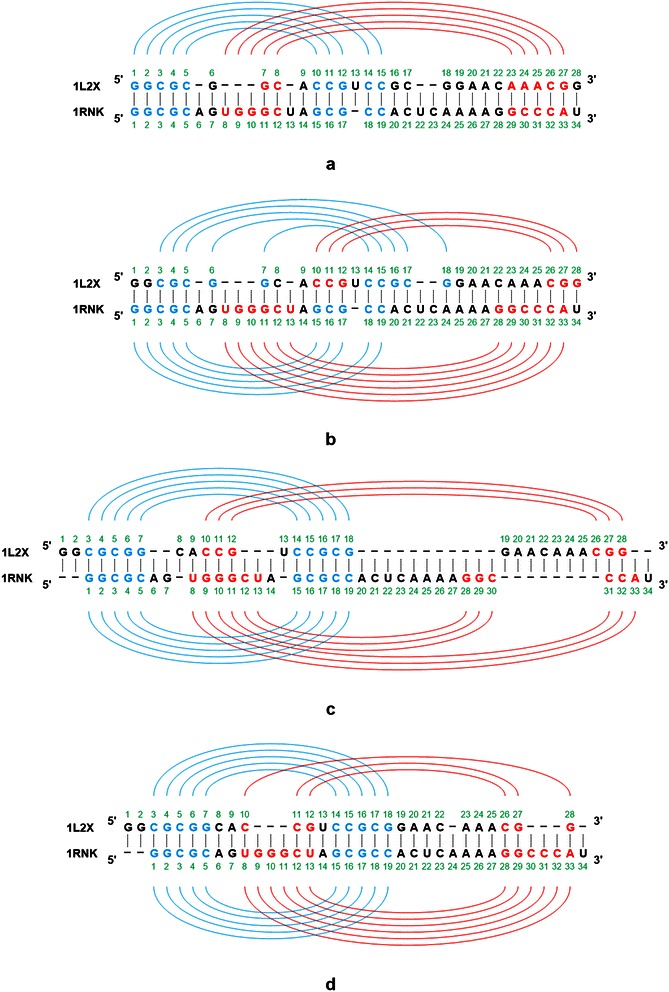
**Example showing base mismatches in an alignment produced by DAFS, R3D Align, and RKalign respectively. (a)** The predicted common secondary structure and the alignment produced by DAFS between two pseudoknot structures with PDB IDs 1L2X and 1RNK respectively. **(b)** The known secondary structures of 1L2X and 1RNK and the alignment produced by DAFS. **(c)** The known secondary structures of 1L2X and 1RNK and the alignment produced by R3D Align. **(d)** The known secondary structures of 1L2X and 1RNK and the alignment produced by RKalign. The base_mismatch ratio of DAFS (R3D Align, RKalign, respectively) is 57.14% (67.39%, 27.78%, respectively), where the base_mismatch ratios are calculated using the known secondary structures. RKalign produces the best alignment with respect to the known secondary structures of 1L2X and 1RNK.

For example, consider Figure 3, which shows the alignment result of DAFS, R3D Align and RKalign respectively on two pseudoknot structures with PDB IDs 1L2X and 1RNK. The base_mismatch ratio of DAFS (R3D Align, RKalign, respectively) is 57.14% (67.39%, 27.78%, respectively). Figure 3(a) shows the predicted common secondary structure and the alignment produced by DAFS. Figure 3(b) shows the known secondary structures of 1L2X and 1RNK and the alignment produced by DAFS where the known secondary structures are used to calculate the base_mismatch ratios. Figure 3(c) shows the alignment obtained from R3D Align and Figure 3(d) shows the alignment obtained from RKalign. It can be seen that the predicted common secondary structure in Figure 3(a) is quite different from the known secondary structure of 1L2X. Refer to Figure 3(b). The base G (G, C, C, A, A and A respectively) at position 1 (2, 8, 22, 23, 24 and 25 respectively) in 1L2X is a single base, which is aligned with the left or right base of some base pair in 1RNK, leading to base mismatches in the alignment. Similarly, the base G (A, C, A and U respectively) at position 7 (20, 21, 24 and 34 respectively) in 1RNK is a single base, which is aligned with the left or right base of some base pair in 1L2X. R3D Align doesn’t align the pseudoknot structures well either, due to the fact that many gaps are involved in the alignment (Figure 3(c)). In this example, RKalign produces the best alignment (Figure 3(d)). It should be pointed out, however, that 3D alignment programs such as R3D Align are general-purpose structure alignment tools capable of comparing two RNA 3D molecules with diverse tertiary motifs, whereas RKalign focuses on secondary structures with pseudoknots only.

In the second experiment, we compared RKalign, CARNA, RNA Sampler and DAFS using the RNA structures in Dataset2. As in the first experiment, we selected 124 pairs of molecules from Dataset2 where the two molecules in a pair belonged to the same pseudoknot type. The average base_mismatch ratio calculated by RKalign for the selected 124 pairs was 35.89%, compared to the average base_mismatch ratio, 81.56%, for all pairs of molecules in Dataset2. We applied each of the four tools to the molecules to produce 124 pairwise alignments. The 124 alignments produced by RKalign can be found in Additional file 3.

**Figure 4:**
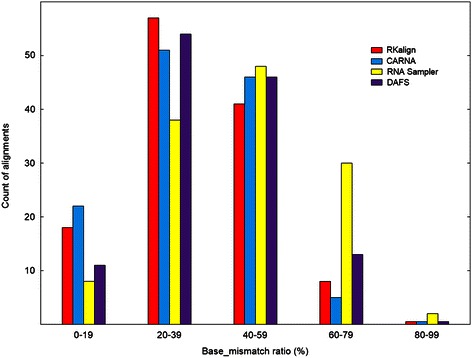
**Histogram for the base_mismatch ratios yielded by RKalign, CARNA, RNA Sampler and DAFS.** Histograms for the base_mismatch ratios of the alignments produced by RKalign, CARNA, RNA Sampler and DAFS respectively on the 124 structure pairs selected from Dataset2. Buckets on the x-axis are defined by equal-width ranges 0 to19, 20 to 39, 40 to 59, 60 to 79, and 80 to 99 (rounded down to the nearest whole number). These histograms show the distribution of the base_mismatch ratios of the alignments produced by the four tools.

**Figure 5:**
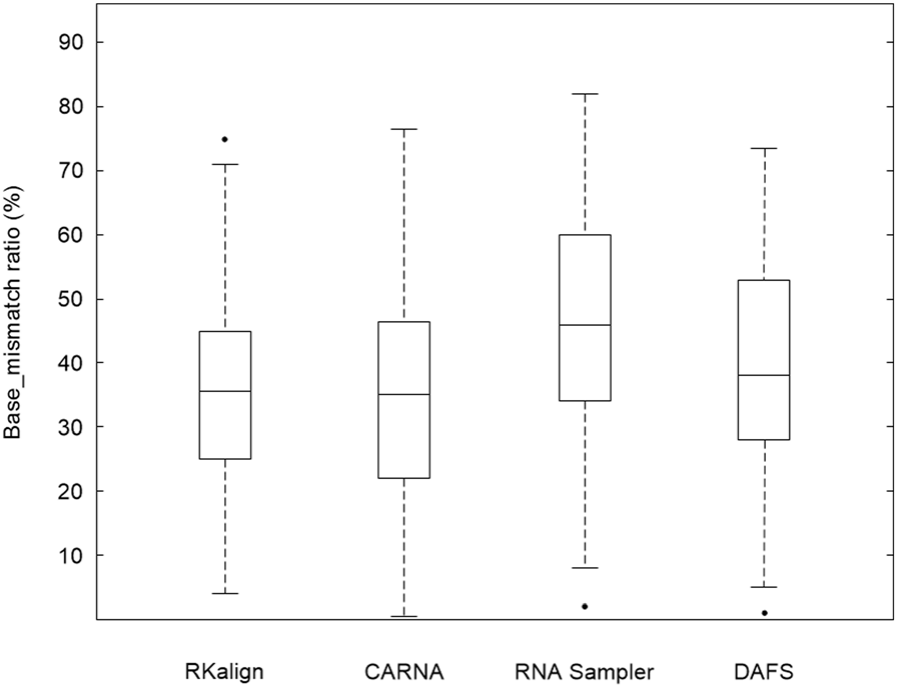
**Boxplot for the base_mismatch ratios of RKalign, CARNA, RNA Sampler and DAFS.** Boxplots for the base_mismatch ratios of the alignments produced by RKalign, CARNA, RNA Sampler and DAFS respectively on the 124 structure pairs selected from Dataset2. The median of the base_mismatch ratios yielded by RKalign (CARNA, RNA Sampler, DAFS, respectively) is 36.31% (35.81%, 45.83%, 39.29%, respectively).

Figure 4 presents histograms for the base_mismatch ratios of the four tools. Figure 5 presents boxplots for the base_mismatch ratios of the four tools. These figures show the distribution of the base_mismatch ratios for the four tools. RKalign and CARNA were not statistically different (Wilcoxon signed rank test, p > 0.05); both tools were significantly better than RNA Sampler and DAFS (Wilcoxon signed rank test, p < 0.05).

Based on the above experimental results, there is no statistically significant difference between RKalign and CARNA in terms of base_mismatch ratios. As described in [4,54], a good pseudoknot alignment has many matched stems and few mismatched stems. In the last experiment, we further compared RKalign with CARNA by examining how they match stems in two pseudoknot structures *A* and *B*. A stem *s*_*A*_ ∈ *A* is said to match a stem *s*_*B*_ ∈ *B* if (i) *s*_*A*_ *,s*_*B*_ are aligned together and they cannot be aligned with other stems, and (ii) for every base pair *x* ∈ *s*_*A*_ and base pair *y* ∈ *s*_*B*_, a base of *x* is aligned with a base of *y* if and only if the other base of *x* is aligned with the other base of *y*; otherwise, there is a stem mismatch between *s*_*A*_ and *s*_*B*_ *.* The stem_mismatch ratio of an alignment *a*_*A,B*_ between structure *A* and structure *B* is defined as (1 – *M*) where *M* is the number of matched stems in *a*_*A,B*_ divided by the total number of stems in *A* and *B*, multiplied by 100%.

**Figure 6:**
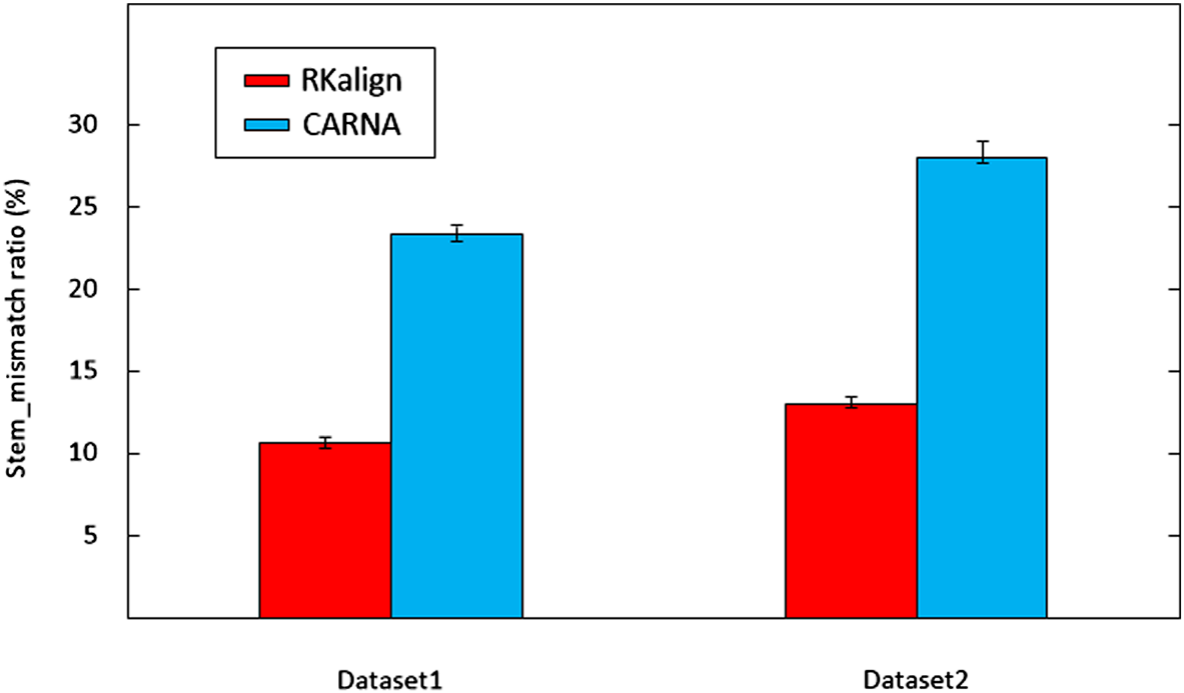
**Comparison of the stem_mismatch ratios yielded by RKalign and CARNA.** Average stem_mismatch ratios of the alignments produced by RKalign and CARNA on the 106 structure pairs selected from Dataset1 and the 124 structure pairs selected from Dataset2 respectively. Error bars are included in the figure. For Dataset1, the average stem_mismatch ratio of RKalign is 10.8% and the average stem_mismatch ratio of CARNA is 23.5%. For Dataset2, the average stem_mismatch ratio of RKalign is 13.1% and the average stem_mismatch ratio of CARNA is 28.9%. RKalign performs significantly better than CARNA in terms of stem_mismatch ratios (Wilcoxon signed rank test, p < 0.05).

**Figure 7:**
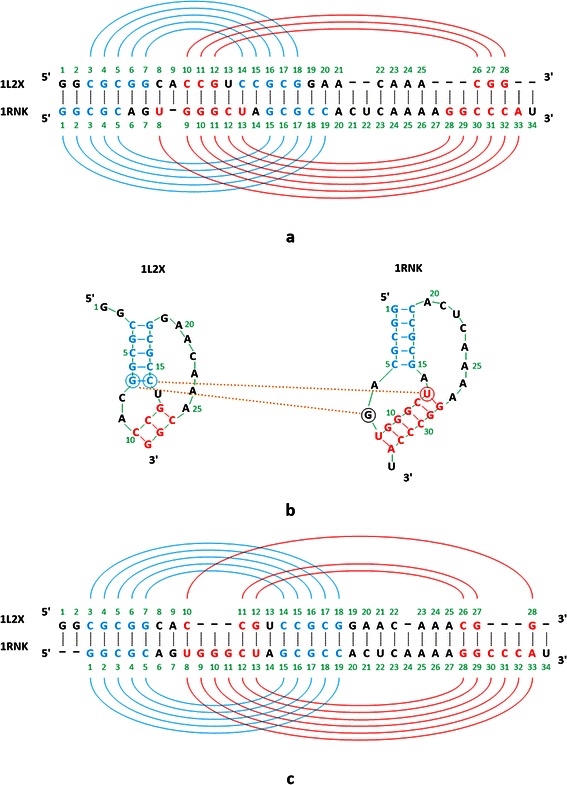
**Example showing mismatched stems in an alignment produced by CARNA. (a)** The alignment of two pseudoknot structures with PDB IDs 1L2X and 1RNK respectively produced by CARNA. **(b)** Illustration of mismatched stems in the alignment produced by CARNA. There is a stem mismatch between the blue stem of 1L2X and the red stem of 1RNK, a situation that is not favored when performing pseudoknot alignment. **(c)** The alignment of 1L2X and 1RNK produced by RKalign where there is no stem mismatch.

Figure 6 shows the average stem_mismatch ratios of RKalign and CARNA obtained by running the tools on Dataset1 and Dataset2 respectively. RKalign was significantly better than CARNA (Wilcoxon signed rank test, p < 0.05). A close look at the alignment results of CARNA reveals why this happens. For instance, consider Figure 7(a), which shows how CARNA aligns the two PDB structures, 1L2X and 1RNK, given in Figure 3. Figure 7(b) illustrates mismatched stems in the alignment in Figure 7(a). Figure 7(c) shows the alignment of the same molecules, 1L2X and 1RNK, produced by RKalign where there is no stem mismatch. Refer to Figure 7(b). In 1L2X, there are two stems, highlighted in blue and red respectively. In 1RNK, there are also two stems, highlighted in blue and red respectively. In 1L2X, the base G at position 7 and the base C at position 14 form a base pair in its blue stem. In 1RNK, the base U at position 13 and the base G at position 28 form a base pair in its red stem.

Now, observe that the base C at position 14 in 1L2X is aligned with the base U at position 13 in 1RNK, but the base G at position 7 in 1L2X is not aligned with the base G at position 28 in 1RNK; instead the base G at position 7 in 1L2X is aligned with the single base G at position 7 in 1RNK. Thus, there is a stem mismatch between the blue stem of 1L2X and the red stem of 1RNK, a situation that is not favored when performing pseudoknot alignment [4,54]. This situation occurs more frequently in CARNA alignment results than in RKalign alignment results. As a consequence, CARNA has much higher stem_mismatch ratios than RKalign.

Comparing Figure 7(a) and Figure 7(c), we also note that the overall alignments produced by CARNA and RKalign are quite different. In Figure 7(a) in which the alignment from CARNA is shown, the base G at position 6 and the base C at position 15 form a base pair in 1L2X. The base A at position 6 in 1RNK is a single base. It can be seen that the base G at position 6 in 1L2X is aligned with the base A at position 6 in 1RNK, i.e., a base pair is aligned with a single base. In addition, in 1L2X the base C at position 14, which is the right base of a base pair, is aligned with the base U at position 13, which is the left base of a base pair in 1RNK. Aligning a base pair with a single base, and aligning the right base of a base pair with the left base of another base pair, occur in CARNA’s output shown in Figure 7(a), but do not occur in RKalign’s output shown in Figure 7(c). On the other hand, there are more gaps in RKalign’s output than in CARNA’s output; specifically there are 10 gaps in RKalign’s output shown in Figure 7(c) compared to 8 gaps in CARNA’s output shown in Figure 7(a).

## Discussion and conclusions

In this paper, we present a novel method (RKalign) for comparing two known RNA pseudoknot structures. The method adopts the partition function methodology to calculate the posterior log-odds scores of the alignments between bases or base pairs of the RNAs with a dynamic programming algorithm. The posterior log-odds scores are then used to calculate the expected accuracy of an alignment between the RNAs. The goal is to find an optimal alignment with the maximum expected accuracy. We present a heuristic to achieve this goal. Experimental results demonstrate the good performance of the proposed RKalign method.

New pseudoknotted structures are found periodically, as exemplified by the recently determined ribosomal CCR5 frameshift pseudoknot [55] and the translational enhancer structures found in the 3′ UTRs of plant viruses [56-59]. It is therefore important to be able to compare these new structures to a database of known pseudoknots to determine the possibility of similar functionality. For example, some of the recently functionally similar pseudoknots found in the 3′ UTRs of plant viruses have been shown to act as translational enhancers and have 3D structures that are similar to tRNAs. Importantly they contain pseudoknots that produce tRNA-like 3D folds, but are not derived from the standard tRNA secondary structure cloverleaf. In addition, these elements have been shown to be important for ribosome binding. RKalign will be useful in performing this kind of database search for structure-function analysis of pseudoknots.

### Extension to multiple alignment

Our pairwise alignment method can be extended to align multiple RNA pseudoknot structures by utilizing a guide tree. Specifically, we treat each structure as a cluster and use the expected accuracy defined in Equation (17) as the measure to determine the similarity of two structures or clusters. Initially, we merge two RNA structures that are most similar into one cluster. Subsequently we merge two clusters that are most similar into a larger cluster using the agglomerative hierarchical clustering algorithm [60], where the similarity of two clusters is calculated by the average linkage algorithm [60].

An alignment of two clusters is actually an alignment of two profiles, where each cluster is treated as a profile. Initially, each profile contains a single RNA pseudoknot structure. As the guide tree grows, a profile may contain multiple RNA pseudoknot structures; more precisely, the profile is a multiple alignment of these RNA structures. A single base of a profile is a column of the profile where the column contains single bases or gaps; a base pair of a profile includes two columns of the profile where the left column contains left bases or gaps and the right column contains corresponding right bases or gaps, and left bases and corresponding right bases form base pairs.

Suppose we want to align profile *A'* and profile *B'*, which amounts to aligning two multiple alignments. Let *R* (*S* respectively) be an RNA pseudoknot structure in profile *A'* (*B'* respectively) and let (*i*, *j*) ((*p*, *q*) respectively) be a base pair of *R* (*S* respectively). Let (*i* ', *j* ') represent a base pair of profile *A'* and let (*p* ', *q* ') represent a base pair of profile *B'*. We use (*i*, *j*) ∈ (*i* ', *j* ') ((*p*, *q*) ∈ (*p* ', *q* ') respectively) to represent that (*i*, *j*) ((*p*, *q*) respectively) occurs in the column(s) of base pair (*i* ', *j* ') ((*p* ', *q* ') respectively) of profile *A'* (*B'* respectively). Equation (18) below shows how to calculate *Prob* ^'^((*i* ^'^, *j* ^'^) ~ (*p* ^'^, *q* ^'^)), which represents the transformed probability of aligning base pair (*i* ', *j* ') of profile *A'* with base pair (*p* ', *q* ') of profile *B'*.

18 $$ Pro{b}^{\mathit{\prime}}\left(\left({i}^{\prime },{j}^{\prime}\right)\sim \left({p}^{\prime },{q}^{\prime}\right)\right)=\frac{{\displaystyle {\sum}_{\left(i,j\right)\in \left({i}^{\prime },{j}^{\prime}\right),\left(p,q\right)\in \left({p}^{\prime },{q}^{\prime}\right)} Prob\left(\left(i,j\right)\sim \left(p,q\right)\right)}}{\left|{A}^{\prime}\right|\left|{B}^{\prime}\right|} $$

Here, *Prob*((*i*, *j*) ~ (*p*, *q*)) is defined in Equation (16), |*A'*| represents the number of RNA pseudoknot structures in profile or cluster *A',* and |*B'*| represents the number of RNA pseudoknot structures in profile or cluster *B'*.

The multiple alignment algorithm can now be summarized as follows. The input of the algorithm is a set *SS* of RNA pseudoknot structures. For every two structures *A* and *B* in *SS*, we calculate their posterior log-odds score matrix as described in the ‘Calculation of posterior log-odds scores’ subsection. After all the posterior log-odds score matrices are calculated, we compute the expected accuracy $\overline{Accu}\left({a}_{A,B}\right)$ as defined in Equation (17) where *a*_*A*,*B*_ is a (sub)optimal alignment, found by the heuristic described in the ‘Pairwise alignment’ subsection, between structure *A* and structure *B*. We use the expected accuracy or similarity values to construct the guide tree for the set *SS*, to determine the order in which two structures or profiles are aligned. To align two profiles *A'* and *B'*, we use the same heuristic as described in the ‘Pairwise alignment’ subsection, with the transformed probabilities *Prob* ' ((*i* ', *j* ') ~ (*p* ', *q* ')) defined in Equation (18) replacing the posterior probabilities *Prob*((*i*, *j*) ~ (*p*, *q*)) of structures *A* and *B* defined in Equation (16). The time complexity of this multiple alignment algorithm is *O*(*k* ^2^ *n* ^4^) where *k* is the number of structures in the alignment and *n* is the maximum of the lengths of the structures; the space complexity of the algorithm is O(*k* ^2^ *n* ^2^).

We tested our algorithm by selecting 30 groups each having 3, 4, or 5 pseudoknot structures of the same type from the datasets used in this study, and by performing multiple alignment in each group. The multiple alignments produced by our algorithm can be found in Additional file 4. We then compared our algorithm with three related methods: CARNA [14], RNA Sampler [11] and DAFS [12]. The base_mismatch ratio of a multiple alignment *MA* is defined as the sum of base_mismatch ratios of all pairs of structures in *MA* divided by the total number of structure pairs in *MA*, multiplied by 100%. The average base_mismatch ratio of RKalign (CARNA, RNA Sampler, DAFS, respectively) was 26.01% (25.79%, 32.15%, 29.23% respectively). RKalign and CARNA were not statistically different (Wilcoxon signed rank test, p > 0.05); the two methods were significantly better than RNA Sampler and DAFS (Wilcoxon signed rank test, p < 0.05).

Both our pairwise alignment and multiple alignment programs are available in the RKalign tool. This tool is capable of accepting as input pseudoknotted RNAs with both sequence (nucleotides or bases) and structure data (base pairs), and producing as output an alignment between the pseudoknotted RNAs. As more and more pseudoknots are revealed, collected and stored in public databases, we anticipate a tool like RKalign will play a significant role in data comparison, annotation, analysis, and retrieval in these databases.

### Comparison with related methods

RKalign is designed to align known RNA pseudoknot structures. A different approach is to simultaneously fold and align RNA sequences without known structures, as adopted by several existing tools [11-13]. When the structure information is not available, this simultaneous folding and alignment approach is the best. However, when pseudoknot structures already exist, RKalign performs significantly better than the existing tools, as observed in our experiments. The reason is that the structures predicted by these tools may not be correct. As a consequence, there are many base mismatches with respect to the known structures in the resulting alignments.

Pseudoknots are part of RNA tertiary motifs [2]. There are 3D alignment programs that can compare RNA tertiary structures including pseudoknots [36,37]. These programs consider the entire RNA 3D structure as a whole, and accept PDB files with 3D coordinates as input. As shown in our experiments, when considering and aligning secondary structures with pseudoknots, RKalign outperforms the 3D alignment programs. It should be noted, however, that the 3D alignment programs are general-purpose structure alignment tools capable of comparing two RNA 3D molecules with diverse tertiary motifs, whereas RKalign deals with secondary structures with pseudoknots only.

While the work reported here focuses on pseudoknot alignment, it can also be applied to RNA secondary structures without pseudoknots. We applied RKalign to 102 pairs of pseudoknot-free structures taken from RNA STARND where the pseudoknot-free structures belonged to Rfam [46] (see Additional file 1: Table S3). We compared RKalign with three other tools: CARNA [14], RNAforester [41] and RSmatch [40]. RNAforester, included in the widely used Vienna RNA package [61], is a versatile RNA structure alignment tool. Like RKalign and CARNA, an option of RNAforester is able to accept as input two RNA molecules with both sequence data (nucleotides or bases) and secondary structure data (base pairs), and produce as output the global alignment of the two molecules. However, a limitation of RNAforester is that the aligned secondary structures cannot contain pseudoknots. RSmatch is similar to RNAforester, sharing the same limitation. Our experimental results showed that the average base_mismatch ratio for RKalign (CARNA, RNAforester, RSmatch, respectively) was 43.52% (42.27%, 35.11%, 39.66%, respectively), indicating RNAforester performed the best. These results are understandable, considering that RKalign is mainly designed for comparing complex pseudoknot structures whereas RNAforester focuses on simpler pseudoknot-free structures.

The work that is most closely related to RKalign is CARNA [14]. Both methods are able to accept as input known pseudoknot structures and produce as output an alignment of the known structures. Our experimental results indicated that the two methods perform well in terms of base_mismatch ratios, though RKalign yields much lower stem_mismatch ratios. It should be pointed out, however, that the comparison with CARNA is not completely fair. The input data of RKalign are restricted to fixed structures, which are structures used in this study. Using CARNA with fixed structures is more or less a mis-use of the tool. The main purpose of CARNA is to align dot-plots, and its scoring is optimized for that data format. Thus, when dot-plots are considered, one should use CARNA. When fixed structures are considered, RKalign is recommended.

### Availability

The latest version of RKalign can be downloaded at: http://bioinformatics.njit.edu/RKalign.

## Additional files

Additional file 1:
**Supplementary information for ‘Effective alignment of RNA pseudoknot structures using partition function posterior log-odds scores’.** **Table S1.** The RNA pseudoknot structures selected from the PDB and RNA STRAND to perform the alignment quality experiments. **Table S2.** The RNA pseudoknot structures selected from PseudoBase to perform the alignment quality experiments. **Table S3.** The RNA pseudoknot-free structures selected from Rfam and RNA STRAND to perform the alignment quality experiments.
Additional file 2:
**The 106 pairwise alignments produced by RKalign on Dataset1.**

Additional file 3:
**The 124 pairwise alignments produced by RKalign on Dataset2.**

Additional file 4:
**The 30 multiple alignments produced by RKalign.**
