# Supplementary material for: Effective alignment of RNA pseudoknot structures using partition function posterior log-odds scores
Source: BMC Bioinformatics. 2015 Feb 6;16:39. doi: 10.1186/s12859-015-0464-9 (PMC4339682; doi:10.1186/s12859-015-0464-9)
Supplement: Additional file 1: — Supplementary information for ‘Effective alignment of RNA pseudoknot structures using partition function posterior log-odds scores’. Table S1. The RNA pseudoknot structures selected from the PDB and RNA STRAND to perform the alignment quality experiments. Table S2. The RNA pseudoknot structures selected from PseudoBase to perform the alignment quality experiments. Table S3. The RNA pseudoknot-free structures selected from Rfam and RNA STRAND to perform the alignment quality experiments. [file 12859_2015_464_MOESM1_ESM.docx]

Supplementary information for ‘Effective alignment of RNA pseudoknot structures using partition function posterior log-odds scores’

Yang Song, Lei Hua, Bruce A. Shapiro, Jason T.L. Wang

S1 Selected RNA pseudoknot structures from the PDB and RNA STRAND

The RNA pseudoknot structures that are selected from the PDB and RNA STRAND are listed below. We use this dataset to evaluate the performance of RKalign, CARNA, RNA Sampler, DAFS, R3D Align and RASS. Each three-dimensional (3D) molecule (e.g. 2AW7) is retrieved from the PDB and used by R3D Align and RASS. The secondary structure of the 3D molecule is obtained with RNAview and stored in RNA STRAND (e.g. [PDB_00935](http://www.rnasoft.ca/strand/show_results.php?molecule_ID=PDB_00935)), which is used by RKalign and CARNA.

Table S1: The RNA pseudoknot structures selected from the PDB and RNA STRAND to perform the alignment quality experiments

| PDB ID | RNA STRAND ID | Molecule Name | RNA Type | Length |
| --- | --- | --- | --- | --- |
| 2AW7 | [PDB_00935](http://www.rnasoft.ca/strand/show_results.php?molecule_ID=PDB_00935) | Crystal structure of the bacterial ribosome from Escherichia coli at 3.5 A resolution | 16S rRNA | 1530 |
| 2I2P | [PDB_01120](http://www.rnasoft.ca/strand/show_results.php?molecule_ID=PDB_01120) | Crystal structure of ribosome with messenger RNA and the anticodon stem-loop of P-site tRNA | 16S rRNA | 1553 |
| 2B57 | [PDB_00944](http://www.rnasoft.ca/strand/show_results.php?molecule_ID=PDB_00944) | Guanine Riboswitch C74U mutant bound to 2,6-diaminopurine | Synthetic RNA | 65 |
| 1FJG | [PDB_00408](http://www.rnasoft.ca/strand/show_results.php?molecule_ID=PDB_00408) | Structure of the Thermus Thermophilus 30S ribosomal subunit in complex with the Antibiotics Streptomycin, Spectinomycin, and Paromomycin | 16S rRNA | 1513 |
| 2FD0 | [PDB_01049](http://www.rnasoft.ca/strand/show_results.php?molecule_ID=PDB_01049) | HIV-1 DIS kissing-loop in complex with lividomycin | Synthetic RNA | 46 |
| 2D19 | [PDB_00988](http://www.rnasoft.ca/strand/show_results.php?molecule_ID=PDB_00988) | Solution RNA structure of loop region of the HIV-1 dimerization initiation site in the kissing-loop dimer | Synthetic RNA | 34 |
| 1Y3O | [PDB_00831](http://www.rnasoft.ca/strand/show_results.php?molecule_ID=PDB_00831) | HIV-1 DIS RNA subtype F- Mn soaked | Synthetic RNA | 46 |
| 3B4C | [PDB_01302](http://www.rnasoft.ca/strand/show_results.php?molecule_ID=PDB_01302) | T. tengcongensis glmS ribozyme bound to glucosamine-6-phosphate and a substrate RNA with a 2'5'-phosphodiester linkage | Other Ribozyme | 139 |
| 1XP7 | [PDB_00816](http://www.rnasoft.ca/strand/show_results.php?molecule_ID=PDB_00816) | HIV-1 subtype F genomic RNA Dimerization Initiation Site | Synthetic RNA | 46 |
| 437D | [PDB_00269](http://www.rnasoft.ca/strand/show_results.php?molecule_ID=PDB_00269) | Crystal structure of an RNA pseudoknot from beet western yellow virus involved in ribosomal frameshifting | Other rRNA | 28 |
| 2G1W | [PDB_01059](http://www.rnasoft.ca/strand/show_results.php?molecule_ID=PDB_01059) | NMR structure of the Aquifex aeolicus tmRNA pseudoknot PK1 | Synthetic RNA | 22 |
| 1L2X | [PDB_00138](http://www.rnasoft.ca/strand/show_results.php?molecule_ID=PDB_00138) | Atomic resolution crystal structure of a viral RNA pseudoknot | Viral & Phage | 28 |
| 3B4A | [PDB_01300](http://www.rnasoft.ca/strand/show_results.php?molecule_ID=PDB_01300) | T. tengcongensis glmS ribozyme with G40A mutation, bound to glucosamine-6-phosphate | Other Ribozyme | 142 |
| 1FFZ | [PDB_00403](http://www.rnasoft.ca/strand/show_results.php?molecule_ID=PDB_00403) | Large ribosomal subunit complexed with R(CC)-Da-Puromycin | Other rRNA | 500 |
| 2TPK | [PDB_00243](http://www.rnasoft.ca/strand/show_results.php?molecule_ID=PDB_00243) | An investigation of the structure of the pseudoknot within gene 32 messenger RNA of Bacteriophage T2 using heteronuclear NMR methods | Viral & Phage | 36 |
| 1KAJ | [PDB_00124](http://www.rnasoft.ca/strand/show_results.php?molecule_ID=PDB_00124) | Conformation of an RNA pseudoknot from mouse mammary tumor virus, NMR, 1 structure | Synthetic RNA | 32 |
| 1VC5 | [PDB_00764](http://www.rnasoft.ca/strand/show_results.php?molecule_ID=PDB_00764) | Crystal structure of the Wild Type Hepatitis Delta Virus Genomic Ribozyme Precursor, in EDTA solution | Other Ribozyme | 70 |
| 1JGQ | [PDB_00486](http://www.rnasoft.ca/strand/show_results.php?molecule_ID=PDB_00486) | The path of messenger RNA through the ribosome | Other RNA | 229 |
| 2FCY | [PDB_01047](http://www.rnasoft.ca/strand/show_results.php?molecule_ID=PDB_01047) | HIV-1 DIS kissing-loop in complex with Neomycin | Synthetic RNA | 46 |
| 1BAU | [PDB_00018](http://www.rnasoft.ca/strand/show_results.php?molecule_ID=PDB_00018) | NMR structure of the dimer initiation complex of HIV-1 Genomic RNA, minimized average structure | Synthetic RNA | 46 |
| 1KPZ | [PDB_00135](http://www.rnasoft.ca/strand/show_results.php?molecule_ID=PDB_00135) | PEMV-1 P1-P2 frameshifting pseudoknot regularized average structure | Synthetic RNA | 28 |
| 1E95 | [PDB_00041](http://www.rnasoft.ca/strand/show_results.php?molecule_ID=PDB_00041) | Solution structure of the pseudoknot of SRV-1 RNA, involved in ribosomal frameshifting | Other RNA | 36 |
| 1DRZ | [PDB_00346](http://www.rnasoft.ca/strand/show_results.php?molecule_ID=PDB_00346) | U1A Spliceosomal Protein/Hepatitis Delta Virus Genomic Ribozyme complex | Other Ribozyme | 72 |
| 1SJ3 | [PDB_00714](http://www.rnasoft.ca/strand/show_results.php?molecule_ID=PDB_00714) | Hepatitis Delta Virus Genomic Ribozyme Precursor, with Mg2+ bound | Other Ribozyme | 73 |
| 2OOM | [PDB_01194](http://www.rnasoft.ca/strand/show_results.php?molecule_ID=PDB_01194) | NMR structure of a kissing complex formed between the TAR RNA element of HIV-1 and a LNA/RNA aptamer | Synthetic RNA | 32 |
| 1JGO | [PDB_00484](http://www.rnasoft.ca/strand/show_results.php?molecule_ID=PDB_00484) | The path of messenger RNA through the ribosome | Other RNA | 232 |
| 2B8S | [PDB_00951](http://www.rnasoft.ca/strand/show_results.php?molecule_ID=PDB_00951) | Structure of HIV-1(MAL) genomic RNA DIS | Synthetic RNA | 46 |
| 1RNK | [PDB_00209](http://www.rnasoft.ca/strand/show_results.php?molecule_ID=PDB_00209) | The structure of an RNA pseudoknot that causes efficient frameshifting in mouse mammary tumor virus | Synthetic RNA | 34 |
| 1SJF | [PDB_00716](http://www.rnasoft.ca/strand/show_results.php?molecule_ID=PDB_00716) | Crystal structure of the Hepatitis Delta Virus Genomic Ribozyme Precursor, with C75U mutation, in Cobalt Hexammine solution | Other Ribozyme | 74 |
| 2AP5 | [PDB_00931](http://www.rnasoft.ca/strand/show_results.php?molecule_ID=PDB_00931) | Solution structure of the C27A ScYLV P1-P2 frameshifting pseudoknot, average structure | Synthetic RNA | 28 |
| 1YG4 | [PDB_00843](http://www.rnasoft.ca/strand/show_results.php?molecule_ID=PDB_00843) | Solution structure of the ScYLV P1-P2 frameshifting pseudoknot, regularized average structure | Synthetic RNA | 28 |
| 1F27 | [PDB_00053](http://www.rnasoft.ca/strand/show_results.php?molecule_ID=PDB_00053) | Crystal structure of a Biotin-Binding RNA pseudoknot | Synthetic RNA | 30 |
| 1KPD | [PDB_00133](http://www.rnasoft.ca/strand/show_results.php?molecule_ID=PDB_00133) | A mutant RNA pseudoknot that promotes ribosomal frameshifting in mouse mammary tumor virus, NMR, minimized average structure | Other rRNA | 32 |
| 1IBK | [PDB_00463](http://www.rnasoft.ca/strand/show_results.php?molecule_ID=PDB_00463) | Structure of the Thermus Thermophilus 30S ribosomal subunit in complex with the antibiotic paromomycin | 16S rRNA | 1512 |
| 2F4X | [PDB_01040](http://www.rnasoft.ca/strand/show_results.php?molecule_ID=PDB_01040) | NMR solution of HIV-1 Lai kissing complex | Synthetic RNA | 48 |
| 1E8O | [PDB_00352](http://www.rnasoft.ca/strand/show_results.php?molecule_ID=PDB_00352) | Core of the ALU domain of the Mammalian SRP | Synthetic RNA | 50 |
| 2NUG | [PDB_01165](http://www.rnasoft.ca/strand/show_results.php?molecule_ID=PDB_01165) | Crystal structure of RNase III from Aquifex aeolicus complexed with ds-RNA at 1.7-Angstrom resolution | Synthetic RNA | 44 |
| 1FG0 | [PDB_00404](http://www.rnasoft.ca/strand/show_results.php?molecule_ID=PDB_00404) | Large ribosomal subunit complexed with A 13 BP Minihelix-Puromycin compound | Other rRNA | 499 |

S2 Selected RNA pseudoknot structures from PseudoBase

The RNA pseudoknot structures that are selected from PseudoBase are listed below. We use this dataset to evaluate the performance of RKalign, CARNA, RNA Sampler and DAFS.

Table S2: The RNA pseudoknot structures selected from PseudoBase to perform the alignment quality experiments

| PKB Number | Abbreviation | Organism | RNA Type | Length |
| --- | --- | --- | --- | --- |
| PKB106 | IBV | infectious bronchitis virus | Viral frameshift | 57 |
| PKB121 | STNV1_PK1 | satellite tobacco necrosis virus 1 | Viral 3 UTR | 26 |
| PKB122 | STNV1_PK2 | satellite tobacco necrosis virus 1 | Viral 3 UTR | 31 |
| PKB123 | STNV1_PK3 | satellite tobacco necrosis virus 1 | Viral 3 UTR | 26 |
| PKB124 | STNV2_PK1 | satellite tobacco necrosis virus 2 | Viral 3 UTR | 29 |
| PKB125 | STNV2_PK2 | satellite tobacco necrosis virus 2 | Viral 3 UTR | 25 |
| PKB126 | STNV2_PK3 | satellite tobacco necrosis virus 2 | Viral 3 UTR | 27 |
| PKB127 | EAV | equine arteritis virus | Viral frameshift | 56 |
| PKB128 | BEV | Berne virus | Viral frameshift | 59 |
| PKB131 | NGF-H1 | - | Aptamers | 48 |
| PKB132 | NGF-L2 | - | Aptamers | 49 |
| PKB133 | NGF-L6 | - | Aptamers | 48 |
| PKB144 | ORSV-S1_PKbulge1 | odontoglossum ringspot virus | Viral tRNA-like | 71 |
| PKB145 | ORSV-S1_PKbulge2 | odontoglossum ringspot virus | Viral tRNA-like | 58 |
| PKB146 | ORSV-S1_PKbulge3 | odontoglossum ringspot virus | Viral tRNA-like | 50 |
| PKB158 | TRV-PSG2_PK1 | tobacco rattle virus, strain PSG | Viral others | 28 |
| PKB159 | TRV-PSG2_PK2 | tobacco rattle virus, strain PSG | Viral others | 25 |
| PKB160 | TRV-PSG2_PK3 | tobacco rattle virus, strain PSG | Viral others | 32 |
| PKB161 | TRV-PSG2_PK4 | tobacco rattle virus, strain PSG | Viral others | 24 |
| PKB162 | TRV-PSG2_PK5 | tobacco rattle virus, strain PSG | Viral others | 35 |
| PKB2 | BWYV | Beet western-yellows virus | Viral ribosomal frameshifting | 50 |
| PKB240 | BChV | beet chlorosis virus | Viral frameshift | 41 |
| PKB254 | SARS-CoV | SARS coronavirus | Viral frameshift | 82 |
| PKB258 | Hs_Ma3 | Homo sapiens | Viral frameshift | 60 |
| PKB3 | EIAV | Equine infectious anemic virus | Viral ribosomal frameshifting | 54 |
| PKB309 | IFNG_PK_B_Taurus | Bos taurus (cow) | mRNA | 145 |
| PKB310 | IFNG_PK_C_familiaris | Canis familiaris (dog) | mRNA | 130 |
| PKB311 | IFNG_PK_C_jacchus | Callitrix jacchus (marmoset) | mRNA | 120 |
| PKB313 | IFNG_PK_S_scrofa | Sus scrofa (pig) | mRNA | 130 |
| PKB346 | KUNV | West Nile virus, Kunijn subtype | Viral frameshift | 75 |
| PKB347 | WNV | West Nile virus | Viral frameshift | 75 |
| PKB348 | JEV | Japanese encephalitis virus | Viral frameshift | 77 |
| PKB350 | ALFV | Alfuy virus | Viral frameshift | 77 |
| PKB4 | FIV | Feline immunodeficiency virus | Viral ribosomal frameshifting | 50 |
| PKB44 | CABYV | cucurbit aphid-borne yellows virus | Viral frameshift | 39 |
| PKB46 | BYDV-NY-RPV | barley yellow dwarf virus | Viral frameshift | 39 |

S3 Selected RNA pseudoknot-free structures from Rfam and RNA STRAND

The RNA pseudoknot-free structures that are selected from Rfam and RNA STRAND are listed below. We use this dataset to evaluate the performance of RKalign, CARNA, RNAforester and RSmatch.

Table S3: The RNA pseudoknot-free structures selected from Rfam and RNA STRAND to perform the alignment quality experiments

| Rfam ID | RNA STRAND ID | Molecule Name | RNA Type | Length |
| --- | --- | --- | --- | --- |
| RF00008 AJ005299.1/335-282 | RFA_00396 | Hammerhead ribozyme (type III), RF00008, AJ005299.1/282-335 | Ham. Ribozyme | 54 |
| RF00008 AJ247113.1/53-134 | RFA_00420 | Hammerhead ribozyme (type III), RF00008, AJ247113.1/134-53 | Ham. Ribozyme | 82 |
| RF00008 AJ247122.1/52-132 | RFA_00423 | Hammerhead ribozyme (type III), RF00008, AJ247122.1/132-52 | Ham. Ribozyme | 81 |
| RF00008 AJ295018.1/1-58 | RFA_00426 | Hammerhead ribozyme (type III), RF00008, AJ295018.1/58-1 | Ham. Ribozyme | 58 |
| RF00008 AJ536619.1/152-206 | RFA_00430 | Hammerhead ribozyme (type III), RF00008, AJ536619.1/206-152 | Ham. Ribozyme | 55 |
| RF00008 Y14700.1/53-133 | RFA_00450 | Hammerhead ribozyme (type III), RF00008, Y14700.1/133-53 | Ham. Ribozyme | 81 |
| RF00019 K01562.1/110-1 | RFA_00582 | Y RNA, RF00019, K01562.1/1-110 | Y RNA | 110 |
| RF00019 K01564.1/77-1 | RFA_00584 | Y RNA, RF00019, K01564.1/1-77 | Y RNA | 77 |
| RF00019 L15431.1/134-36 | RFA_00585 | Y RNA, RF00019, L15431.1/36-134 | Y RNA | 99 |
| RF00019 L15432.1/124-36 | RFA_00586 | Y RNA, RF00019, L15432.1/36-124 | Y RNA | 89 |
| RF00019 L27537.1/96-1 | RFA_00588 | Y RNA, RF00019, L27537.1/1-96 | Y RNA | 96 |
| RF00019 X57566.1/93-1 | RFA_00595 | Y RNA, RF00019, X57566.1/1-93 | Y RNA | 93 |
| RF00025 AF399707.1/2345-2181 | RFA_00603 | Ciliate telomerase RNA, RF00025, AF399707.1/2181-2345 | Cili. Telo. RNA | 165 |
| RF00025 AJ132318.1/175-1 | RFA_00605 | Ciliate telomerase RNA, RF00025, AJ132318.1/1-175 | Cili. Telo. RNA | 175 |
| RF00025 M33461.1/346-140 | RFA_00606 | Ciliate telomerase RNA, RF00025, M33461.1/140-346 | Cili. Telo. RNA | 207 |
| RF00025 U10565.1/238-50 | RFA_00608 | Ciliate telomerase RNA, RF00025, U10566.1/72-260 | Cili. Telo. RNA | 189 |
| RF00025 U22353.1/206-53 | RFA_00614 | Ciliate telomerase RNA, RF00025, U22353.1/53-206 | Cili. Telo. RNA | 154 |
| RF00025 U45435.1/283-69 | RFA_00618 | Ciliate telomerase RNA, RF00025, U45435.1/69-283 | Cili. Telo. RNA | 215 |
| RF00109 AB169770.1/1705-1641 | RFA_00640 | Vimentin 3 prime UTR protein-binding region, RF00109, AB169770.1/1641-1705 | Cis-reg. element | 65 |
| RF00109 AF447708.1/1826-1750 | RFA_00642 | Vimentin 3 prime UTR protein-binding region, RF00109, AF447708.1/1750-1826 | Cis-reg. element | 77 |
| RF00109 BC053115.1/1568-1499 | RFA_00645 | Vimentin 3 prime UTR protein-binding region, RF00109, BC053115.1/1499-1568 | Cis-reg. element | 70 |
| RF00109 M26251.1/2000-1935 | RFA_00652 | Vimentin 3 prime UTR protein-binding region, RF00109, M26251.1/1935-2000 | Cis-reg. element | 66 |
| RF00163 X15620.1/62-19 | RFA_00715 | Hammerhead ribozyme (type I), RF00163, X15620.1/19-62 | Ham. Ribozyme | 44 |
| RF00163 Z69686.1/390-342 | RFA_00720 | Hammerhead ribozyme (type I), RF00163, Z69686.1/342-390 | Ham. Ribozyme | 49 |
| RF00524 U13031.1/1644-1423 | RFA_00810 | R2 RNA element, RF00524, U13031.1/1423-1644 | Other RNA | 222 |
| RF00524 U13033.1/1658-1423 | RFA_00812 | R2 RNA element, RF00524, U13033.1/1423-1658 | Other RNA | 236 |
| RF00524 U81985.1/709-508 | RFA_00818 | R2 RNA element, RF00524, U81985.1/508-709 | Other RNA | 202 |
| RF00524 X51967.1/3585-3349 | RFA_00819 | R2 RNA element, RF00524, X51967.1/3349-3585 | Other RNA | 237 |
